# Tailored Crystallization Dynamics for Efficient and Stable DMSO‐Free Tin Perovskite Solar Cells

**DOI:** 10.1002/advs.202501311

**Published:** 2025-05-23

**Authors:** Shengnan Zuo, Alexander Tarasov, Lennart Frohloff, Karunanantharajah Prashanthan, Florian Ruske, Mailis Lounasvuori, Chiara Frasca, André Dallmann, Fengshuo Zu, Florian Mathies, Florian Scheler, Noor Titan Putri Hartono, Guixiang Li, Jinzhao Li, Maxim Simmonds, Wenhui Li, Norbert Koch, Steve Albrecht, Meng Li, Eva Unger, Mahmoud Hussein Aldanmasy, Artem Musiienko, Antonio Abate

**Affiliations:** ^1^ Helmholtz‐Zentrum Berlin für Materialien und Energie GmbH Department Active Materials and Interfaces for Stable Perovskite Solar Cells (SE‐AMIP) Kekuléstraße 5 12489 Berlin Germany; ^2^ Helmholtz‐Zentrum Berlin für Materialien und Energie GmbH Department Solution Processing of Hybrid Materials & Devices (SE‐ALM) Kekuléstraße 5 12489 Berlin Germany; ^3^ Helmholtz‐Zentrum Berlin für Materialien und Energie GmbH Department Perovskite Tandem Solar Cells (SE‐APET) Kekuléstraße 5 12489 Berlin Germany; ^4^ Helmholtz‐Zentrum Berlin für Materialien und Energie GmbH Department Spins in Energy Conversion and Quantum Information Science (SE‐ASPIN) Hahn‐Meitner‐Platz 1 14109 Berlin Germany; ^5^ Helmholtz‐Zentrum Berlin für Materialien und Energie GmbH Young Investigator Group Nanoscale Solid‐Liquid Interfaces Hahn‐Meitner Platz 1 14109 Berlin Germany; ^6^ Helmholtz‐Zentrum Berlin für Materialien und Energie GmbH Young Investigator Group Robotized Material and Photovoltaic Engineering Kekuléstraße 5 12489 Berlin Germany; ^7^ Humboldt University of Berlin Department of Physics Brook‐Taylor‐Straße 6–BT6 12489 Berlin Germany; ^8^ Humboldt University of Berlin Institude of Chemistry Brook‐Taylor‐Str. 2 12489 Berlin Germany; ^9^ Department of Physics University of Jaffna 40000 Jaffna Sri Lanka; ^10^ Key Laboratory for Special Functional Materials of Ministry of Education Henan University 475004 Kaifeng P. R. China; ^11^ Institute of Chemical Research of Catalonia (ICIQ‐CERCA) Avda. Països Catalans, 16 43007 Tarragona Spain

**Keywords:** crystallization, DMSO‐free, in situ PL, tin perovskites

## Abstract

Tin perovskite solar cells are emerging as a sustainable lead‐free alternative in thin film photovoltaics. DMSO‐free processed tin perovskites are gaining interest due to the detrimental effects of DMSO on tin oxidation. However, replacing DMSO with other solvents remains challenging due to the accelerated crystallization dynamics in non‐DMSO systems. In this study, the crystallization process in a DMSO‐free solvent system is regulated by managing the transition from the sol‐gel phase to the solid film. Specifically, piperazine dihydriodide (PDAI) and 4‐*tert*‐butylpyridine (tBP) are utilized to coordinately tune the colloidal chemistry through forming large pre‐nucleation clusters in perovskite ink, further, facilitating the film formation process. By combining tBP and PDAI, a controllable crystallization rate is achieved as evidenced by in situ photoluminescence (PL) measurement during spin‐coating. As a result, tin perovskite films show high crystallinity and improved microstructure. Devices treated with tBP+PDAI exhibit a champion power conversion efficiency of 7.8% and excellent stability without observable degradation for over 3000 h stored in the N_2_ glovebox. These findings advance understanding and managing crystallization in DMSO‐free solvents processed tin perovskite solar cells.

## Graphical abstract

In this work, the authors investigated the crystallization process of tin perovskites in a DMSO‐free solvent system using in situ PL technique. The authors discovered that a synergistic approach effectively facilitates the formation of high‐quality DMSO‐free tin perovskite films by modulating the emergence of crystallites and the aggregation process. As a result, they achieved efficient and stable tin devices without DMSO.

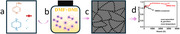

## 1 Introduction

Perovskite solar cells (PSCs) have achieved remarkable efficiency in recent years. With a state‐of‐the‐art power conversion efficiency (PCE) of over 26%, PSCs are competitive with silicon‐based technologies such as mono and polycrystalline silicon.^[^ ^1^ ^]^ However, these high efficiencies are delivered using lead‐based perovskites, which would pose some environmental concerns.^[^ ^2^ ^]^ Developing environmentally friendly lead‐free perovskite technology is becoming fundamental for further industrialization. The perovskite community started looking for a replacement for lead as early as 2012.^[^ ^3^ ^]^ Lead alternatives are limited due to atomic size and electronic band structure constraints. Tin (Sn) is an environmentally friendly alternative to lead in perovskite materials due to its size and electronic distribution similarity.^[^ ^4^, ^5^, ^6^ ^]^ In addition, tin perovskites showed an ideal bandgap of 1.35 eV and comparable optoelectronic properties to lead perovskites, enabling them to achieve higher efficiency according to Shockley–Queisser theory.^[^ ^7^, ^8^, ^9^ ^]^

Noticeably, tin PSCs appear to meet a bottleneck, as they have shown limited improvements in efficiency in recent years and suffer from issues such as irreproducibility of results compared to their lead‐based counterparts.^[^ ^10^, ^11^, ^12^, ^13^, ^14^, ^15^ ^]^ The main causes behind this include the easy oxidation of Sn^2+^ to Sn^4+^, the fast crystallization process and the misaligned energy bands between perovskite films and contact layers.^[^ ^16^, ^17^, ^18^, ^19^ ^]^ Latest, Y. Shi et al. have achieved a certified PCE of 15.7% for Formamidinium‐based tin triiodide (FASnI_3_) PSCs by adjusting energy‐level alignment between the perovskite and electron transport layers, leading to 0.974 V of open‐circuit voltage.^[^ ^20^ ^]^ However, enhancing device stability is still underway.

Dimethyl sulfoxide (DMSO) is a commonly used solvent for processing highly efficient tin PSCs due to its strong coordination with Sn^2+^.^[^ ^11^, ^21^ ^]^ Nevertheless, DMSO can oxidize Sn^2+^ during the annealing process. Moreover, the residual DMSO can lead to the formation of Sn^4+^, which initiates self‐p‐doping and trap states, further affect the long‐term device stability.^[^ ^22^, ^23^, ^24^, ^25^ ^]^ Potentially, DMSO‐free solvent systems could mitigate the Sn^2+^ oxidation induced by DMSO. The alternative solvents basically accelerate the crystallization process, resulting in low‐quality films with increased pinholes and defects.^[^ ^26^, ^27^ ^]^ Therefore, it's crucial to modulate the crystallization process to grow high‐quality Sn perovskite films in a DMSO‐free solvent system. However, precisely controlling crystallization kinetic remains under‐explored in DMSO‐free solvents processed tin PSCs.

In this work, we combine 4‐*tert*‐butylpyridine (tBP) and piperazine dihydriodide (PDAI) as co‐additives in perovskite suspension with dimethylformamide (DMF) and 1,3‐dimethyl‐2‐imidazolidinone (DMI) to modulate the colloidal chemistry and further control the crystallization kinetics. To the best of our knowledge, both tBP and PDAI have been individually studied by peers in the context of tin perovskite crystallization. Our previous work demonstrated that tBP can control the crystallization process as a co‐solvent by interacting with SnI_x_ units in a DMSO‐free solvent system.^[^ ^28^ ^]^ X. Meng et al. proposed that PDAI facilitates the formation of pre‐nucleation clusters with reduced Gibbs energy barriers through a non‐classical nucleation mechanism in a DMSO‐contained system.^[^ ^29^ ^]^ Nevertheless, a comprehensive understanding of the transition from “sol‐gel phase to solid film” remains unclear, particularly in a non‐DMSO solvent system. Herein, we leverage the advantage of tBP and PDAI to investigate the modulation process in DMSO‐free tin perovskites. The film formation process is monitored using the in situ photoluminescence (PL) technique during spin‐coating. It shows that the combination of tBP and PDAI gives a synergistic effect, promoting high‐quality film formation through regulated crystallization dynamics. As a result, devices with tBP+PDAI achieve a champion power conversion efficiency (PCE) of 7.8%, significantly outperforming inactive control devices. Notably, the unencapsulated devices demonstrate superior stability, with Sn^2+^ antioxidation properties and maintain performance over 3000 h of storage in the N_2_ glovebox without any noticeable decay.

## 2 Results and Discussion

**Figure 1:**
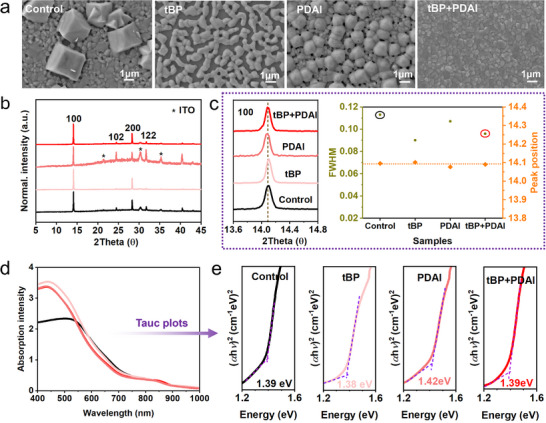
Microstructure and optical measurements of "Control" and treated samples. a) Top‐viewed SEM images of perovskite films: “Control”, “tBP”, “PDAI” and “tBP+PDAI”. b) Normalized XRD patterns. c) Enlarged XRD peak of (100) plane (left); FWHM and positions of (100) crystal plane statistics (right), fitted by Gaussian function. d) UV–vis–NIR absorption spectra. e) Tauc plots fitted of “Control”, “tBP”, “PDAI” and “tBP+PDAI”.

To directly observe the impact of additives on film formation, we prepared four different perovskite inks. These included the stock precursor with FASnI_3_ dissolved in a DMF: DMI solvent mixture (used as the “Control”), as well as three variants with the addition of 10 vol% tBP (“tBP” precursor), 1 mol% PDAI (“PDAI” precursor), and a combination of tBP and PDAI (“tBP+PDAI” precursor) to the stock precursor, representing separate conditions. Top‐view scanning electron microscope (SEM) was examined for the films' microstructures under these four conditions, as shown in **Figures** **1**a and S1 (Supporting Information). We can observe a significant difference among these four perovskite films. For the “Control” film, large micrometre‐sized grains are inserted into the continuous nanometre‐grained film with disconnected grain boundaries. Film with tBP presents a more homogeneous microstructure but has disconnected domains, like “mesh film”. The PDAI‐treated film shows continuous but inhomogeneous grains and rough surface. The above three film microstructure cases could be attributed to uncontrollable crystallization rates with fast solidification during the perovskite film formation.^[^ ^30^ ^]^ Film containing tBP+PDAI exhibits a neat and compact microstructure, indicating the synergistic effect of tBP and PDAI in forming well‐constructed perovskite films. Considering the effect of ink wettability on film continuity, we checked the contact angles of these four inks on PEDOT: PSS/ITO substrates. We found that the wettability of various inks did not significantly affect film microstructure due to similar contact angles from Figure S2 (Supporting Information). In addition, we analysed the grain size distribution from different microstructures. As shown in Figure S3 (Supporting Information), we noticed that sample “tBP+PDAI” shows a smaller grain size distribution with a range of 150–800 nm compared to the other three types, which may be attributed to well‐controlled crystallization dynamics.

Furthermore, X‐ray diffraction (XRD) and UV–vis–NIR absorption spectra were measured to analyze the crystallinity and optical properties of the prepared films, as shown in Figure 1b,d, respectively. As compared to fitted parameters of (100) crystal planes, the “tBP+PDAI” film shows enhanced crystal quality from a lower FWHM value than the “Control” film in Figure 1c and Table S1 (Supporting Information). Interestingly, we observed the lowest FWHM for “tBP” film amongst the four samples while the SEM image of the tBP sample exhibits porous microstructure and non‐consistent grain sizes. This phenomenon might be ascribed to the inhomogeneity of grain distributions, as well as “Control” and “PDAI” samples. Meanwhile, both the “Control” and “tBP+PDAI” films exhibit nearly identical peak positions at the (100) plane, indicating that additional tBP+PDAI does not disrupt the crystal structure. However, (100) positions of films treated with tBP and PDAI individually show slight shifts compared to “Control” films. The absorption spectrum in Figure 1d shows enhanced light absorption after additive treatment compared to “Control” film in the range of 400 nm‐ 550 nm. Besides, Tauc plots fitted from the absorption spectrum were exhibited in Figure 1e. We found that the bandgap of the “tBP+PDAI” film (1.39 eV) is almost consistent with that of the “Control” film (1.39 eV). The bandgaps of films with PDAI and tBP individually are 1.38 and 1.42 eV, respectively. Given the negligible changes for the “tBP+PDAI” film, along with a slight shift for individual tBP and PDAI films compared to the “Control” film, as observed from XRD and absorption, we conclude that tBP+PDAI added together effectively neutralizes the effects on the crystal lattice, alleviating and balancing lattice stress.

**Figure 2:**
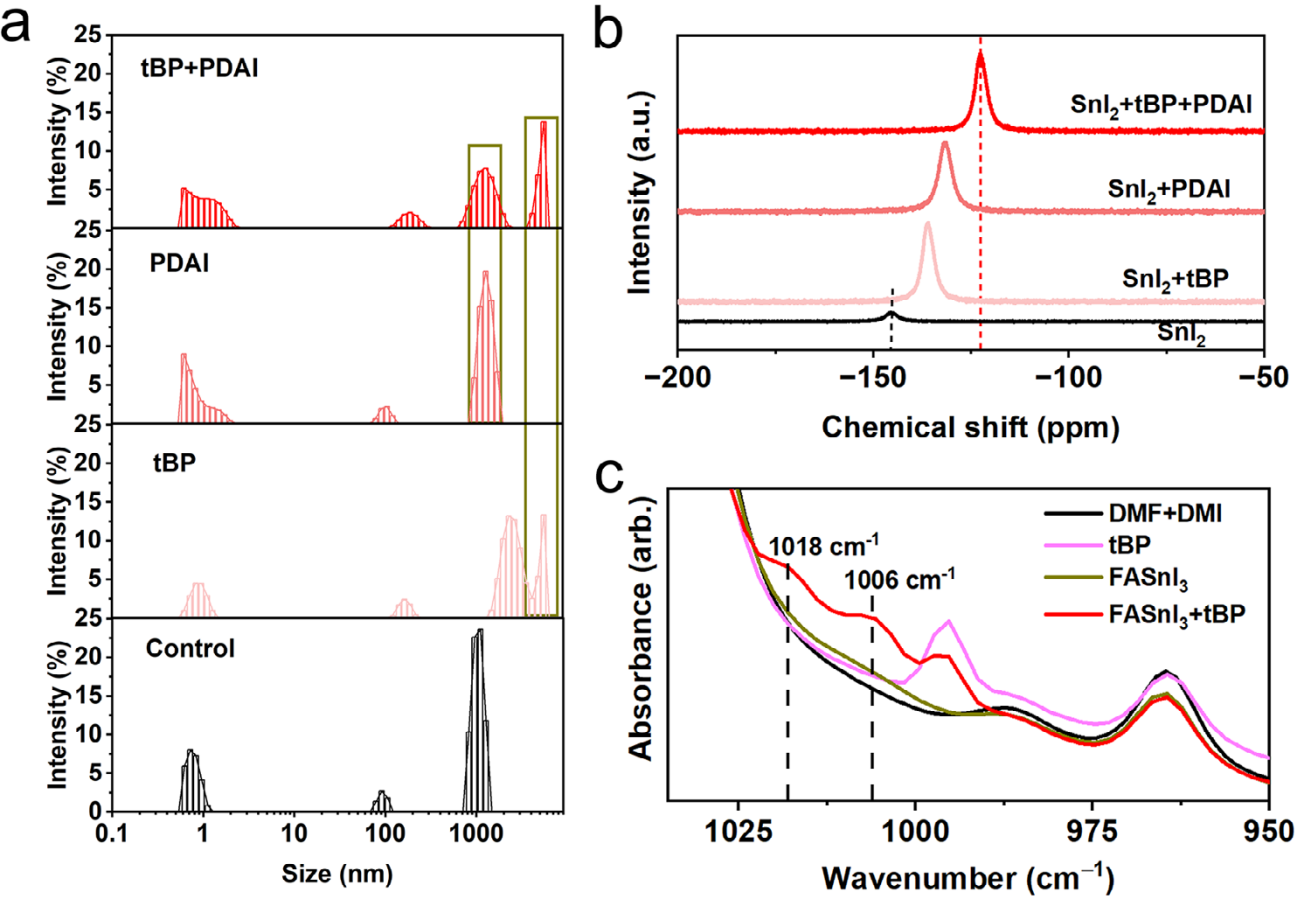
Chemical properties of perovskite inks. a) Colloidal size distribution measured by dynamic light scattering for perovskite inks: “Control”, “tBP”, “PDAI” and “tBP+PDAI”. b) ^119^Sn‐NMR signals measured for SnI_2_, SnI_2_+tBP, SnI_2_+PDAI and SnI_2_+tBP+PDAI dissolved in DMF‐d_7_ at 1 M concentration. c) FTIR spectra of FASnI_3_ inks with and without tBP, pure DMF+DMI solvents with and without tBP.

As reported, the colloidal chemistry of perovskite ink can significantly influence the formation of perovskite film.^[^ ^31^ ^]^ We then performed dynamic light scattering (DLS) measurement to study the colloidal size distribution, as shown in **Figure** **2**a. Compared to the “Control” ink, the colloidal sizes in the “tBP,” “PDAI,” and “tBP+PDAI” inks are significantly larger, likely due to interactions between the Sn compounds and the respective additives. The presence of large aggregates is believed to facilitate the film formation process as pre‐nucleation clusters.^[^ ^29^ ^]^ Notably, the “tBP+PDAI” ink exhibits two distinct regions of large size distribution, which correspond to the individual effects of tBP and PDAI, indicating a combined influence from both additives. To confirm the interaction between the Sn compound and additives, ^119^Sn‐NMR and FTIR measurements were conducted. As shown in Figure 2b, the Sn (II) resonance displays a downfield chemical shift in the presence of tBP, PDAI and tBP+PDAI, indicating strong coordination between Sn and the additives. Furthermore, the “tBP+PDAI” ink exhibits a more pronounced chemical shift, suggesting a synergistically enhanced interaction between Sn and the combined additives. It should be noted that the interaction between Sn compounds and PDAI could not be verified by FTIR measurement, due to the low concentration of PDAI in the optimized precursors and the absence of vibrational modes associated with PDAI. (in Figure S4, Supporting Information). However, we believe that strong bonding exists between Sn and PDAI, as supported by our NMR results and previously reported literature.^[^ ^29^ ^]^ As for tBP, the most intense ring stretching band at 1600 cm^−1^ overlaps with the strong solvent bands, thus we focus instead on the weaker ring breathing mode around 1000 cm^−1^, which has been reported to blueshift upon coordination.^[^ ^32^ ^]^ The FTIR spectrum of tBP in the solvent shows the ring breathing mode at 996 cm^−1^ as shown in Figure 2c. When FASnI_3_ is added to the ink, this mode becomes less intense and two new bands can be observed at 1006 and 1018 cm^−1^. The band at 1006 cm^−1^ is attributed to the blue‐shifted ring breathing mode, and the band at 1018 cm^−1^ is assigned to the trigonal symmetry ring stretching mode that becomes more intense upon coordination, in agreement with previous reports.^[^ ^32^, ^33^, ^34^ ^]^

**Figure 3:**
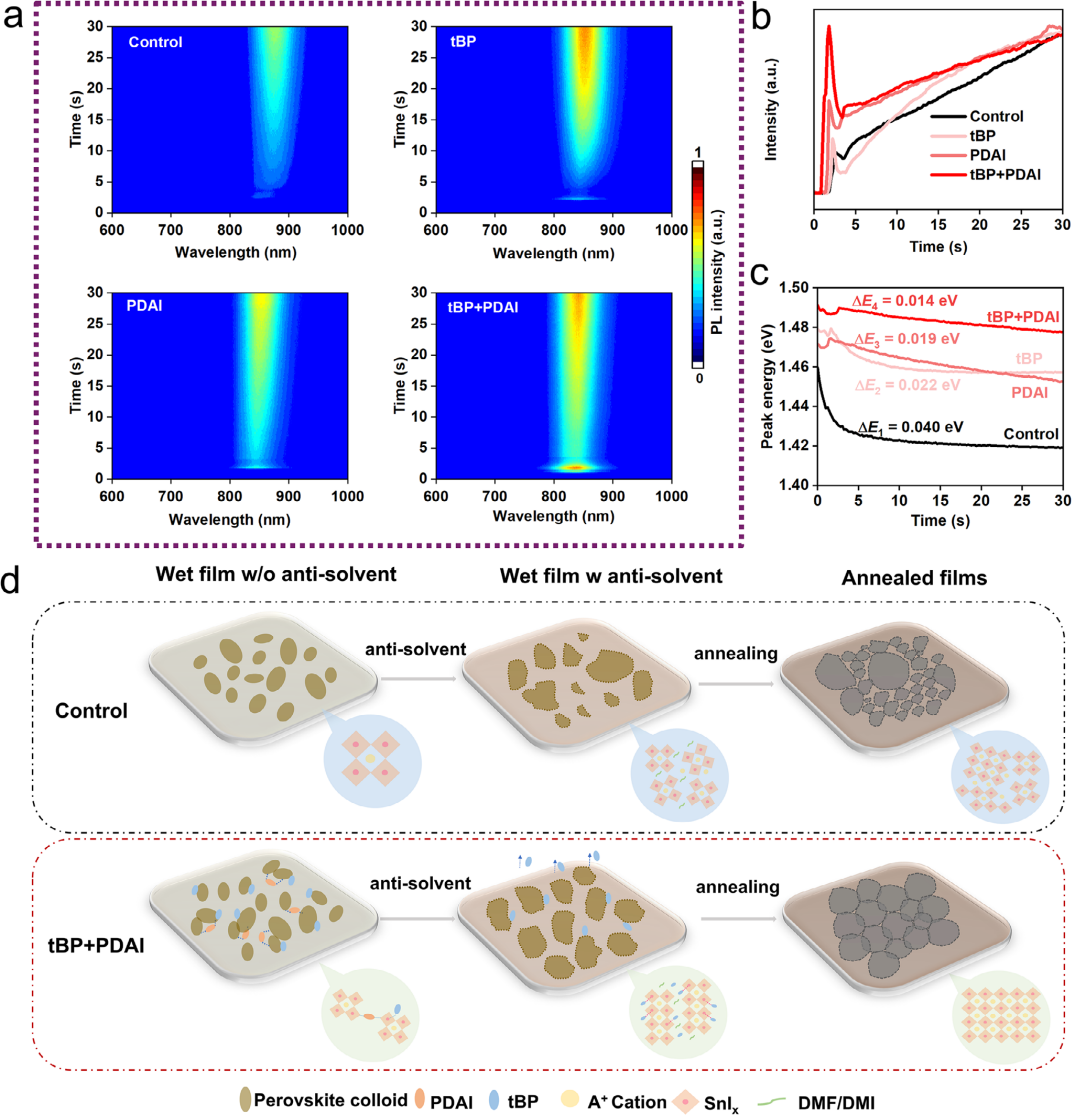
Crystallization kinetic study by in situ PL and the assumed schematic. a) Normalized heat maps of the captured in situ PL spectra of “Control”, “tBP", “PDAI” and “tBP+PDAI" films during spin coating, y‐axis initials at antisolvent dripping. The extracted values of b) normalized PL intensity and c) PL peak energy from PL spectra, x‐axis initials at antisolvent dripping (note that the time point for anti‐solvent dripping might vary in milliseconds due to human error). d) Schematic diagram of the proposed crystallization mechanism for “Control” and “tBP+PDAI” treated films.

For a comprehensive analysis of the crystallization process of tin perovskites, in situ photoluminescence (PL) was characterized to track the evolution of PL‐emissive species during the spin‐coating process. The spectra in the form of heatmaps for all samples are presented in **Figure** **3**a, and the extracted data by applying Gaussian fitting from PL spectra are shown in Figure 3b,c.

PL signal was first detected when antisolvent was applied at ≈13 s after starting the spin‐coating process. The “tBP+PDAI” sample demonstrates the highest intensity of all samples at the time of the antisolvent dripping, indicating a high density of aggregates emerged. This is more apparent from the extracted PL intensity in Figure 3b with all samples except “Control” showing a similar trend‐an intensity maximum followed by an abrupt intensity drop and a “dip” straight after. This is an indication of the rapid emergence of crystallites upon dripping antisolvent and the “dissolution” process.^[^ ^35^ ^]^ After that, the PL intensity increases almost linearly for all samples signifying further “crystallite aggregation”, in accordance with the literature.^[^ ^35^, ^36^ ^]^ The PL intensity diminishes in the following order: tBP+PDAI > PDAI > tBP > Control, suggesting that the mixture of tBP and PDAI has a synergistic influence on the process. Switching attention to the extracted PL peak energies (Figure 3c), all samples with additive reveal that crystallization started around 1.48 eV, and the values slightly red‐shifted (0.014, 0.019, and 0.022 eV for “tBP+PDAI”, “PDAI”, and “tBP”, respectively) over the course of the measurement. This is quite in contrast with the “Control” sample, where a starting value of 1.46 eV exponentially red‐shifted toward the final value of 1.42 eV, rendering a 0.04 eV difference. The redshifts are related to the “poly‐crystallites growth” process in the wet film, thus suggesting the slowest growth rate for “tBP+PDAI” among these four types.^[^ ^36^ ^]^ Overall, it is supported that the use of tBP and PDAI additives promotes the precipitation upon anti‐solvent dripping and slows crystallite growth from sol‐gel phases to solid films compared to additive‐free solvent mixtures, and the effect is most profound with the tBP+PDAI mixture.

Regarding the above results, we propose a moderate crystallization mechanism in the presence of tBP+PDAI, driven by colloidal interactions, as depicted in Figure 3d. First, the “Control” perovskite suspension with distinct colloids is deposited on the substrate. The antisolvent dripping causes fast saturation, leading to the precipitation of the perovskite crystallites without regulation. Sequentially, disconnected domains from random and unoriented nano‐assembled [Sn_x_I_6x_] units are formed, concluding from poor film microstructure and the low in situ PL intensity. As a result, inhomogeneous perovskite film is formed after annealing. However, perovskite suspension with tBP+PDAI contains large pre‐nucleation clusters with reduced critical Gibbs free energy in a non‐classical nucleation pathway.^[^ ^29^, ^37^ ^]^ Subsequently, antisolvent dripping induces a high density of well‐distributed crystallites due to a reduced energy barrier. Benefiting from the synergistic effect of tBP and PDAI, [Sn_x_I_6x_] nano‐assembles are slowly and orderly arranged into matured crystal grains. Therefore, the annealed perovskite film is compact and homogeneous with high‐quality crystal grains.

**Figure 4:**
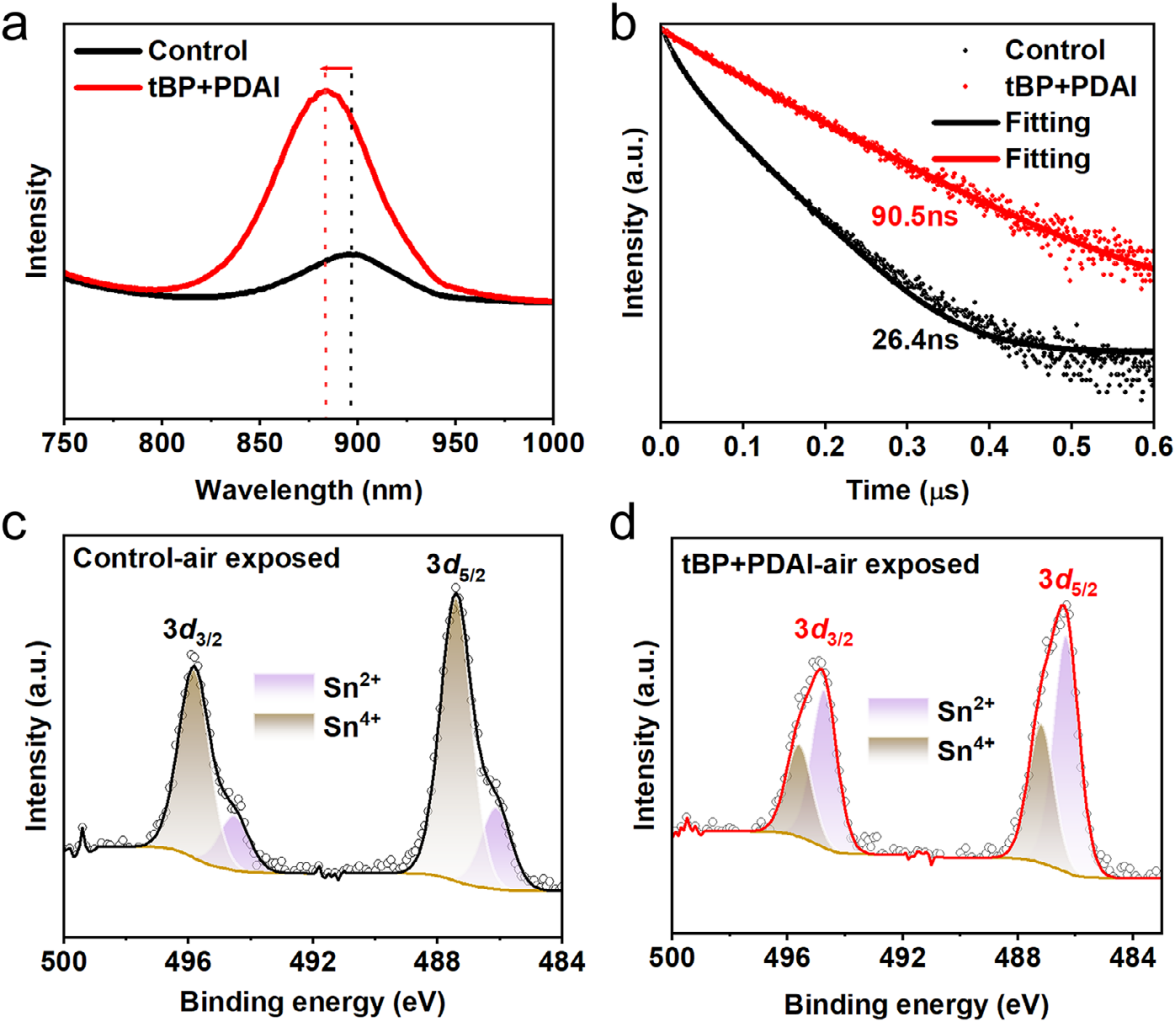
Electronical and charge transport properties of annealed perovskite films “Control” and “tBP+PDAI”. a) Steady‐state PL spectra of perovskite films on glass substrates. b) trPL decay of “Control” and “tBP+PDAI” films deposited on glass substrates. XPS spectra of Sn 3*d* orbitals after 1 h of air‐exposed c) “Control” and d) “tBP+PDAI” films.

To further investigate the film quality of annealed “Control” and “tBP+PDAI” samples, steady‐state PL and time‐resolved PL (trPL) measurements were conducted. **Figure** **4**a shows a higher PL intensity of the tBP+PDAI treated film than the “Control” film, reflecting the enhanced radiative recombination in the film with tBP+PDAI. Besides, the peak position of the “tBP+PDAI” film is blue‐shifted by 20 meV compared with the “Control”, which could be attributed to suppressed defects concentration and reduced non‐radiative recombination due to improved film quality.^[^ ^38^ ^]^ The trPL results were presented in Figure 4b, and the data fitted by a biexponential equation were summarized in Table S2 (Supporting Information). We found that tBP+PDAI can prolong the average carrier lifetime of perovskite film, which is increased to 90 ns from 26 ns of the “Control”, implying that tBP+PDAI can suppress the defects formation^[^ ^38^, ^39^ ^]^ during the crystallization process firmly, consistent with steady‐state PL results. We also examined the antioxidant ability of Sn by air exposing “Control” and “tBP+PDAI” films in the air for 1 h using X‐ray Photoelectron Spectroscopy (XPS), as shown in Figure 4c,d. Sn^2+^ still dominates in “tBP+PDAI” film with a Sn^2+^/Sn^4+^ ratio of 1.7 compared to 0.3 of Sn^2+^/Sn^4+^ in “Control” film, indicating that “tBP+PDAI” film performs great advantage in antioxidant stability even compared with the referenced literature.^[^ ^40^ ^]^ The ratio of Sn^2+^/Sn^4+^ for fresh “tBP+PDAI” films (5.7) and fresh “Control” films (3.2) are shown in Figure S6 and Table S3 (Supporting Information). The antioxidant stability is beneficial for obtaining highly stable tin perovskite devices.

**Figure 5:**
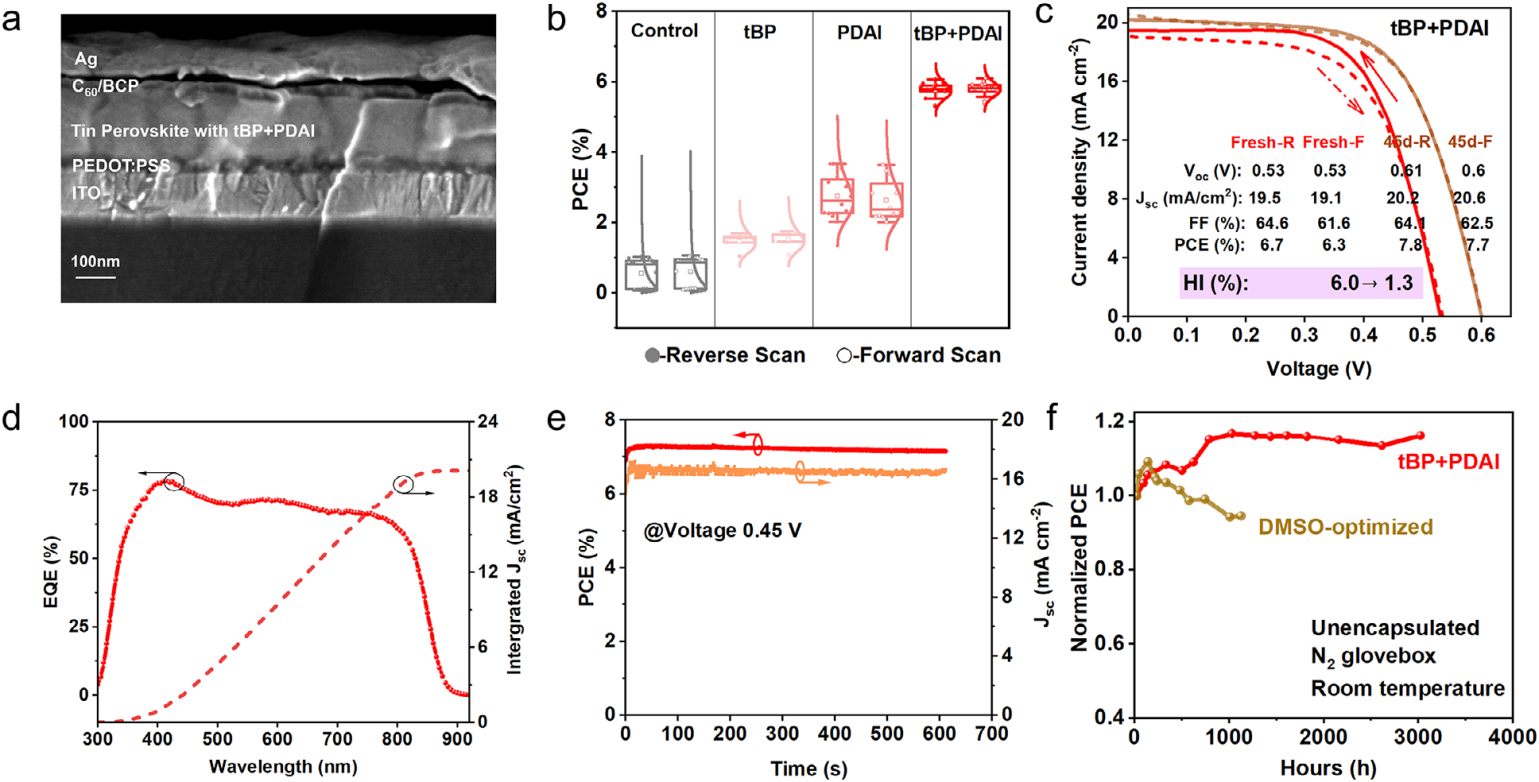
Photovoltaic performance of devices. a) SEM cross‐section of the full device: glass/ITO/PEDOT: PSS/Tin perovskite/C_60_/BCP/Ag. b) Statistical PCEs of “Control”, “tBP", “PDAI”, and “tBP+PDAI” devices. c) Best *J*–*V* curves with reverse and forward scans of fresh devices and aged devices stored in N_2_ glovebox for 45 days, measured under simulated AM1.5G solar illumination at 100 mW cm^−2^. d) EQE and integrated *J_sc_* of the aged champion device. e) Stabilized power output under simulated AM1.5G solar illumination at 100 mW cm^−2^ for 600 s. f) Shelf stability of unencapsulated optimized DMSO and “tBP+PDAI” devices in N_2_ glovebox for over 3000 h.

To assess the impact of tBP+PDAI on device performance, we fabricated solar cell devices with p‐i‐n structure glass/ITO/PEDOT: PSS/perovskite/C_60_/BCP/Ag. We examined the cross‐section of the full device by SEM in **Figure** **5**a. The thickness of the “tBP+PDAI” tin perovskite layer is estimated to be 160 nm. We also demonstrated the statistical efficiencies by employing these four conditions of perovskites in Figure 5b. The content optimization of tBP and PDAI in perovskite suspension are shown in Figures S7 and S8 (Supporting Information). We can observe that “Control” devices exhibit PCEs below 1% due to their unfavourable film microstructure and defects. Devices with tBP and PDAI individually exhibit increased performance. Promisingly, devices with tBP+PDAI present the most enhanced efficiencies with narrower distribution due to improved open‐circuit voltage (*V_oc_*) and Fill Factor (FF), as shown in Figure S9 (Supporting Information). From *J*–*V* curves in Figure 5c, the champion PCE of fresh “tBP+PDAI” device is 6.7% with *V_oc_* of 0.53 V; short‐circuit current density (*J_sc_*) of 19.5 mA cm^−2^; FF of 64.6%. The PCE increased to 7.8% after 45 days (≈1000 h) of storage in the N_2_ glovebox at room temperature, mainly due to an increase in *V_oc_* that reached 0.61 V while *J_sc_* slightly increased to 20.2 mA cm^−2^. The rise in *V_oc_* could be attributed to the subsequent slow defects passivation effect reported in the literature.^[^ ^41^ ^]^ Moreover, the hysteresis in *J*–*V* curve from forward scan (FS) and reverse scan (RS) was decreased from 6% to 1.3% after ageing (Hysteresis Index (HI) is defined as $\mathrm{HI}=PCE_{RS}-PCE_{FS}/PCE_{RS}$).^[^ ^42^ ^]^ The integrated current density of 20.1 mA cm^−2^ from external quantum efficiency (EQE) for the aged device in Figure 5d was consistent with *J_sc_* (20.2 mA cm^−2^) from the sun solar simulator, validating the values obtained for the PCE. Furthermore, device stability was also studied to evaluate its behaviour under operation. Stabilized PCE under continuous light illumination at maximum power point for 600 s showed stable output in Figure 5e. Moreover, unencapsulated devices stored in an N_2_ glovebox for over 3000 h show extremely excellent shelf stability without observable decay as shown in Figure 5f, which exhibited supreme promise in DMSO‐free tin perovskite solar cells. We also performed FASnI_3_ perovskite devices with PDAI and tBP as additives in a DMSO solvent system. There was a 5% decay of efficiencies for DMSO‐processed unencapsulated devices stored in the N_2_ glovebox after 47 days (≈1100 h). DMSO‐free processed devices are up‐and‐coming in stabilizing tin PSCs fabrication. Notably, the thickness of perovskite film leaves room for further exploration of device improvements. Our approach provides a comprehensive understanding of modulating the crystallization of tin perovskites, specifically in a DMSO‐free solvent system.

## 3 Conclusion

The crystallization modulation in DMSO‐free tin PSCs is essential for improving the device's performance. We tuned the crystallization in DMF and DMI solvent systems by modulating the colloidal interactions with two functional additives of PDAI and tBP. Both tBP and PDAI can promote the emergence of crystallites by forming pre‐nucleation clusters in perovskite ink and slowing down the growth of crystallites during the film formation process. The synergistic effect of tBP and PDAI results in improved film crystallinity, suppressed defect formation, extended charge carrier lifetime and enhanced anti‐oxidation ability. Benefiting from these, we significantly enhanced the performance of DMSO‐free tin PSCs, with a champion PCE of 7.8%. The devices also exhibit exceptional long‐term stability, maintaining their performance for over 3000 h in an N_2_ glovebox, compared to 5% decay after over 1000 h for DMSO‐processed tin devices. This approach shows promise in narrowing the efficiency gap between DMSO‐free solvents and DMSO‐contained tin PSCs. Furthermore, considering the improvement in PL properties, our strategy has great potential to be used in other tin‐based optoelectronic devices such as LEDs, sensors, and transistors.

## 4 Experimental Section

Experimental details are provided in the Supporting Information.

## Conflict of Interest

The authors declare no conflict of interest.

## Supporting information



Supporting Information

Supplemental Table 1

Supplemental Table 2

## Data Availability

The data that support the findings of this study are available in the supplementary material of this article.

## References

[1] S. Liu , J. Li , W. Xiao , R. Chen , Z. Sun , Y. Zhang , X. Lei , S. Hu , M. Kober‐Czerny , J. Wang , F. Ren , Q. Zhou , H. Raza , Y. Gao , Y. Ji , S. Li , H. Li , L. Qiu , W. Huang , Y. Zhao , B. Xu , Z. Liu , H. J. Snaith , N.‐G. Park , W. Chen , Nature 2024, 632, 536. 38925147 10.1038/s41586-024-07723-3

[2] J. Li , H.‐L. Cao , W.‐B. Jiao , Q. Wang , M. Wei , I. Cantone , J. Lü , A. Abate , Nat. Commun. 2020, 11, 310. 31964862 10.1038/s41467-019-13910-y PMC6974608

[3] Z. Chen , J. J. Wang , Y. Ren , C. Yu , K. Shum , Appl. Phys. Lett. 2012, 101, 093901.

[4] A. Abate , Joule 2017, 1, 659.

[5] F. Hao , C. C. Stoumpos , R. P. H. Chang , M. G. Kanatzidis , J. Am. Chem. Soc. 2014, 136, 8094. 24823301 10.1021/ja5033259

[6] L. Ma , F. Hao , C. C. Stoumpos , B. T. Phelan , M. R. Wasielewski , M. G. Kanatzidis , J. Am. Chem. Soc. 2016, 138, 14750. 27750426 10.1021/jacs.6b09257

[7] L. Rao , X. Meng , S. Xiao , Z. Xing , Q. Fu , H. Wang , C. Gong , T. Hu , X. Hu , R. Guo , Y. Chen , Angew. Chem. Int. Ed. Engl. 2021, 60, 14693. 33835645 10.1002/anie.202104201

[8] C. C. Stoumpos , C. D. Malliakas , M. G. Kanatzidis , Inorg. Chem. 2013, 52, 9019. 23834108 10.1021/ic401215x

[9] G. Tang , Z. Xiao , J. Hong , J. Phys. Chem. Lett. 2019, 10, 6688. 31608644 10.1021/acs.jpclett.9b02530

[10] E. Hou , J. Chen , J. Luo , Y. Fan , C. Sun , Y. Ding , P. Xu , H. Zhang , S. Cheng , X. Zhao , L. Xie , J. Yan , C. Tian , Z. Wei , Angew. Chem. Int. Ed. Engl. 2024, 63, 202402775. 10.1002/anie.202402775 38468414

[11] X. Jiang , H. Li , Q. Zhou , Q. Wei , M. Wei , L. Jiang , Z. Wang , Z. Peng , F. Wang , Z. Zang , K. Xu , Y. Hou , S. Teale , W. Zhou , R. Si , X. Gao , E. H. Sargent , Z. Ning , J. Am. Chem. Soc. 2021, 143, 10970. 34196528 10.1021/jacs.1c03032

[12] B. Li , X. Wu , H. Zhang , S. Zhang , Z. Li , D. Gao , C. Zhang , M. Chen , S. Xiao , A. K. Y. Jen , S. Yang , Z. Zhu , Adv. Funct. Mater. 2022, 32, 2205870.

[13] H. Li , B. Chang , L. Wang , Z. Wang , L. Pan , Y. Wu , Z. Liu , L. Yin , ACS Energy Lett. 2022, 7, 3889.

[14] B. B. Yu , Z. Chen , Y. Zhu , Y. Wang , B. Han , G. Chen , X. Zhang , Z. Du , Z. He , Adv. Mater. 2021, 33, 2102055. 10.1002/adma.202102055 34296476

[15] F. Yang , R. Zhu , Z. Zhang , Z. Su , W. Zuo , B. He , M. H. Aldamasy , Y. Jia , G. Li , X. Gao , Z. Li , M. Saliba , A. Abate , M. Li , Adv. Mater. 2024, 36, 2308655. 10.1002/adma.202308655 37884347

[16] J.‐J. Cao , Y.‐H. Lou , K.‐L. Wang , Z.‐K. Wang , J. Mater. Chem. C 2022, 10, 7423.

[17] G. Li , Z. Su , M. Li , F. Yang , M. H. Aldamasy , J. Pascual , F. Yang , H. Liu , W. Zuo , D. Di Girolamo , Z. Iqbal , G. Nasti , A. Dallmann , X. Gao , Z. Wang , M. Saliba , A. Abate , Adv. Energy Mater. 2021, 11, 2101539.

[18] L. Lanzetta , T. Webb , N. Zibouche , X. Liang , D. Ding , G. Min , R. J. E. Westbrook , B. Gaggio , T. J. Macdonald , M. S. Islam , S. A. Haque , Nat. Commun. 2021, 12, 2853. 33990560 10.1038/s41467-021-22864-z PMC8121806

[19] Q.‐D. Ou , C. Li , Q.‐K. Wang , Y.‐Q. Li , J.‐X. Tang , Adv. Mater. Interfaces 2017, 4, 1600694.

[20] Y. Shi , Z. Zhu , D. Miao , Y. Ding , Q. Mi , ACS Energy Lett. 2024, 9, 1895.

[21] W. S. Yang , J. H. Noh , N. J. Jeon , Y. C. Kim , S. Ryu , J. Seo , S. I. Seok , Science 2015, 348, 1234. 25999372 10.1126/science.aaa9272

[22] M. I. Saidaminov , I. Spanopoulos , J. Abed , W. Ke , J. Wicks , M. G. Kanatzidis , E. H. Sargent , ACS Energy Lett. 2020, 5, 1153.

[23] J. Pascual , G. Nasti , M. H. Aldamasy , J. A. Smith , M. Flatken , N. Phung , D. Di Girolamo , S.‐H. Turren‐Cruz , M. Li , A. Dallmann , R. Avolio , A. Abate , Mater. Adv. 2020, 1, 1066.

[24] T. Hossain , S. Joy , K. Draffen , R. Bright , S. Johnson , K. R. Graham , ACS Appl. Energy Mater. 2023, 6, 12334.

[25] S. Tian , G. Li , R. C. Turnell‐Ritson , Z. Fei , A. Bornet , M. K. Nazeeruddin , P. J. Dyson , Angew. Chem. Int. Ed. Engl. 2024, 63, 202407193. 10.1002/anie.202407193 38744679

[26] C. Liu , Y. B. Cheng , Z. Ge , Chem. Soc. Rev. 2020, 49, 1653. 32134426 10.1039/c9cs00711c

[27] J. Pascual , D. Di Girolamo , M. A. Flatken , M. H. Aldamasy , G. Li , M. Li , A. Abate , Chemistry 2022, 28, 202103919. 10.1002/chem.202103919 PMC9302133 34878203

[28] G. Nasti , M. H. Aldamasy , M. A. Flatken , P. Musto , P. Matczak , A. Dallmann , A. Hoell , A. Musiienko , H. Hempel , E. Aktas , D. Di Girolamo , J. Pascual , G. Li , M. Li , L. V. Mercaldo , P. D. Veneri , A. Abate , ACS Energy Lett. 2022, 7, 3197. 36277134 10.1021/acsenergylett.2c01749 PMC9578040

[29] X. Meng , Y. Li , Y. Qu , H. Chen , N. Jiang , M. Li , D. J. Xue , J. S. Hu , H. Huang , S. Yang , Angew. Chem. Int. Ed. Engl. 2021, 60, 3693. 33174357 10.1002/anie.202012280

[30] M. Yin , H. Yao , H. Qiu , C. Wu , M. Zhang , F. Hao , Adv. Funct. Mater. 2024, 34, 2404792.

[31] H. Zheng , G. Liu , Y. Wang , F. Chen , X. Dong , C. Wang , C. Wu , L. Yang , X. Ren , L. Yang , X. Pan , Z. Huang , Adv. Funct. Mater. 2024, 34, 2401546.

[32] C. Shi , S. Dai , K. Wang , X. Pan , F. Kong , L. Hu , Vib. Spectrosc. 2005, 39, 99.

[33] L. J. Cao , A. Y. Li , H. B. Ji , L. Xu , Y. Zhang , J. Mol. Struct. 2010, 959, 80.

[34] S. Yurdakul , M. Bahat , J. Mol. Struct. 1997, 412, 6.

[35] T. Huang , S. Tan , S. Nuryyeva , I. Yavuz , F. Babbe , Y. Zhao , M. Abdelsamie , M. H. Weber , R. Wang , K. N. Houk , C. M. Sutter‐Fella , Y. Yang , Sci. Adv. 2021, 7, abj1799. 10.1126/sciadv.abj1799 PMC8580316 34757790

[36] T. B. Song , Z. Yuan , M. Mori , F. Motiwala , G. Segev , E. Masquelier , C. V. Stan , J. L. Slack , N. Tamura , C. M. Sutter‐Fella , Adv. Funct. Mater. 2019, 30, 1908337.

[37] M. Jung , S. G. Ji , G. Kim , S. I. Seok , Chem. Soc. Rev. 2019, 48, 2011. 30604792 10.1039/c8cs00656c

[38] D. W. de Quilettes , S. M. Vorpahl , S. D. Stranks , H. Nagaoka , G. E. Eperon , M. E. Ziffer , H. J. Snaith , D. S. Ginger , Science 2015, 348, 683. 25931446 10.1126/science.aaa5333

[39] M. Stolterfoht , C. M. Wolff , J. A. Márquez , S. Zhang , C. J. Hages , D. Rothhardt , S. Albrecht , P. L. Burn , P. Meredith , T. Unold , D. Neher , Nat. Energy 2018, 3, 847.

[40] B. Chang , B. Li , Z. Wang , H. Li , L. Wang , L. Pan , Z. Li , L. Yin , Adv. Funct. Mater. 2021, 32, 2107710.

[41] E. Jokar , C.‐H. Chien , O. l. Amir Fathi , M. Rameez , Y.‐H. Chang , E. W.‐G. Diau , Energy Environ. Sci. 2018, 11, 2353.

[42] M. Minbashi , E. Yazdani , Sci. Rep. 2022, 12, 14916. 36050358 10.1038/s41598-022-19194-5 PMC9436975

