# Supplementary material for: Tailored Crystallization Dynamics for Efficient and Stable DMSO‐Free Tin Perovskite Solar Cells
Source: Adv Sci (Weinh). 2025 May 23;12(29):e01311. doi: 10.1002/advs.202501311 (PMC12362759; doi:10.1002/advs.202501311)
Supplement: Supplementary file 1 — Supporting Information [file ADVS-12-e01311-s001.docx]

**Supporting information**

**Tailored Crystallization Dynamics for Efficient and Stable DMSO-Free Tin Perovskite Solar Cells**

*Shengnan Zuo, Alexander Tarasov, Lennart Frohloff, Karunanantharajah Prashanthan, Florian Ruske,* *Mailis Lounasvuori,* *Chiara Frasca,* *André Dallmann,* *Fengshuo Zu, Florian Mathies, Florian Scheler,* *Noor Titan Putri Hartono, Guixiang Li, Jinzhao Li, Maxim Simmonds, Wenhui Li,* *Norbert Koch,* *Steve Albrecht, Meng Li, Eva Unger, Mahmoud Hussein Aldanmasy^*^, Artem Musiienko^*^, Antonio Abate^*^*

**Experimental section:**

Materials:

Formamidinium iodide (FAI, 99.99%) was purchased from Dyenamo; Phenethylammonium iodide (PEAI) was bought from Xi’an Yuri Solar Co., Ltd; Tin (II) iodide (SnI_2_, 99.99%), Tin(II) fluoride (SnF2, 99%), Dimethylformamide (DMF), Dimethyl sulfoxide (DMSO); Piperazinium diiodide (PDAI) was obtained from Luminance Technologies Ltd.; Buckminsterfullerene (C_60_, 99.95%, OE) were provided by Creaphys GmbH; 1,3-Dimethyl-2-imidazolidinone (DMI, >99%) was bought from TCI; Bathocuproine (BCP, 99.8%) was acquired from Ossila. Silver shots (Ag, 2-3 mm, 99.999%) was purchased from Alfa Aesar. Mucasol solution was obtained from Schülke & Mayr GmbH; Ethanol and isopropanol (IPA) were received from VWR International Ltd. Patterned indium tin oxide (ITO) glass slides (OLED grade, 10 Ohm/sq) were purchased from Automatic Research GmbH. PEDOT: PSS polymer dispersion in water (CLEVIOS™ HTL Solar) was purchased from Heraeus.

Perovskite precursor solutions:

SnI_2_ and FAI were scaled as molar ratio 1:1 and dissoved in 1mL DMF: DMI=6:1 mixture solvents at nominal concentration of 0.9 mmol/mL. Extra 10 mol% SnF_2_ and 8 mmol PEAI was added into 1mL precursor solution simultaneously, mixing in the shaker at room temperature for around 3 hours. tBP (50uL, 100uL, 150uL respectively) was added into 1mL precursor solution as tBP precursor; PDAI (3mg/mL≈1%, 6mg/mL≈2%, 10mg/mL≈3%) was introduced to 1mL precursor solution as PDAI precursor; tBP (100uL) and PDAI (1%) were added into 1mL precursor solution as tBP+PDAI precursor. It’s noted that tBP should be added before half hour when the solution is going to be used. All the solutions were filtered with 0.2 μm filter before use. For DMSO perovskite precursor solution, we used 100% DMSO to substitute DMF and DMI mixture as solvents.

Device fabrication:

Patterned ITO glasses were cleaned by using 2% Mucasol/water solution, deionized water, aceton and IPA under ultra-sonication for 15min at 40°C sequentially. After that, ITO substrates were dried by flowing nitrogen (N_2_) gun and placed into UV-ozone cleaner for 15min. Then, 150 uL PEDOT: PSS solution was spread as hole transport layer (HTL) at 4500 rpm for 45s on top of glass/ITO, then annealed at 140°C for 30min in the air. Noted that PEDOT: PSS dispersion was filtered by a 0.45 μm filter before use. After this step, glass/ITO/PEDOT: PSS was transferred into N_2_ glovebox quickly preventing the negative effect exposing in the air. Afterwards, 100uL perovskite precursor solution was spin-coated on top of glass/ITO/PEDOT: PSS at 4000 rpm for 45s, and 150 uL p-Xylene as anti-solvent was dripped onto perovskite film after 13s during spin-coating. After that, the substrates were transferred to a hot plate immediately at 100°C for 10min. For DMSO perovskite films fabrication, we used 10s, 5000 rpm and 50s, 5000 rpm as the spin-coating program, 150 uL Chlorobenzene as anti-solvent was dripped at 40s after spinning. Noted that the tin perovskite precursor solution was filtered by a 0.2μm PTFE filter before use in the N_2_ glovebox. Subsequently, glass/ITO/PEDOT: PSS/tin perovskite was moved into evaporator for depositing C_60_, BCP and Ag. Finally, 23nm C_60_, 8nm BCP and 100nm Ag were evaporated on the active area 0.16 cm^2^.

**Characterization**

**The J–V curves** were measured using a digital source meter (Keithley 2400) under AM 1.5 G conditions. Light intensity was calibrated by a silicon cell KG3 before measuring. External quantum efficiency (**EQE)** was performed by using a Xenon lamp (Newport 300W), optical chopper (Oriel instrument) and a Stanford Research SR830 Lock-in amplifier, monitored and evaluated in the TracQ-Basic software. X-ray diffraction (**XRD)** measurement was operated by Bruker D8 X-ray diffractometer, with Cu Kα radiation (λ= 0.15405 nm), X-ray generator setting 40kV and 40mA, samples were protected in N_2_ atmosphere within a half-sphered dome. Scanning electron microscopy (**SEM)** was characterized by ZEISS GEMINI 2 and captured by inLens detector. **UV-Vis-NIR** absorption spectrum was performed by Cary Series UV-Vis-NIR spectrophotometer from Agilent Technologies under double beam mode. Bandgaps are calculated from Tauc plots of UV-Vis-NIR absorption spectrum using the equation: (*αhν*)^2^ = *A* (*hν* − *E*_g_). α is the absorption coefficient, calculated from α=(1/d)ln(1/T) where d is the film thickness and T is the Tansmission. **Steady state Photoluminescence (PL)** spectrum was measured by a home-built setup using QEPro High-Performance spectrometer (Ocean Insight) and LuQY Control software, laser wavelength of 520nm as an excitation wavelength. The PL setup was installed with reflectance lenses to integrate the reflected light. **Time-resolved Photoluminescence (PL)** measurements were carried out on a home-built confocal PL setup utilising a 90:10 transmission: reflection beamsplitter to separate the excitation and detection paths. Excitation was by a 700nm diode laser (IB-705-B laser head with Taiko driver, Picoquant) operated in pulsed mode with repetition rate of 50kHz. The laser beam was passed through a 700-10nm band pass filter (FF01-700/13-25, Semrock) and the laser beam energy was set via a graded ND filter plate to 500nW average power. The spotsize diameter was calculated to be 150um in diameter. Photoluminescence detection was by a silicon singlephoton avalanche diode (Laser Components COUNT-50). The photoluminescence signal was selected by optical filters: a 715nm longpass filter (FF01-715/LP-25, Semrock) for the perovskite PL signal. PL count rate was recorded by TimeHarp260 Nano time-correlated single photon counting module (Picoquant). **In-situ PL measurement** was carried out in N_2_ glovebox using QEPro High-Performance spectrometer, data was analysed by a in-house written software SpectraAnalyzer. **The x-ray photoemission spectra (XPS)** were acquired with a JEOL JPS-9030 with a vacuum base pressure of 3x10^-9^ mbar using monochromated Al Kα radiation (hν = 1486.6 eV) for excitation. The spectra were recorded under an enmission angle of ~10° and pass energy 20 eV, resulting in a setup resolution of 820 meV as determined from the FWHM of the Ag 3d_5/2_ peak. **Liquid-State ^119^Sn NMR spectra** were all acquired on a Bruker AV4 600 MHz equipped with a room-temperature TBO probe head. Typically, a sweep width of 507.8 ppm was used and 64k points were acquired, resulting in a total acquisition time of 288.36 ms. The center frequency was set at 0 ppm. We used a 30 ° pulse to minimize the recycle delay down to 2 s. The number of scans was 256. **Dynamic light scattering (DLS)** was measured by Malvern setup and data was acquired by Zetasizer software, temperature was set to 20 °C. **Contact angle** was measured by KRÜSS, and report was obtained from KRÜSS ADVANCE software. **FTIR spectra** were acquired with a Bruker Alpha FTIR spectrometer equipped with a diamond ATR accessory and a room-temperature DLaTGS detector. The entire spectrometer was placed inside a N_2_ glovebox with oxygen content <0.1 ppm. Liquid samples were measured by dropping 10 microlitres of the sample onto the ATR crystal. Solid PDAI was first ground in a mortar to reduce and homogenise the particle size before a small amount was placed on the ATR crystal. Intimate contact with the ATR crystal was achieved with a pressure clamp. A freshly cleaned ATR crystal was used as the background in all cases. 256 scans were co-added for both background and samples.

**Figures**


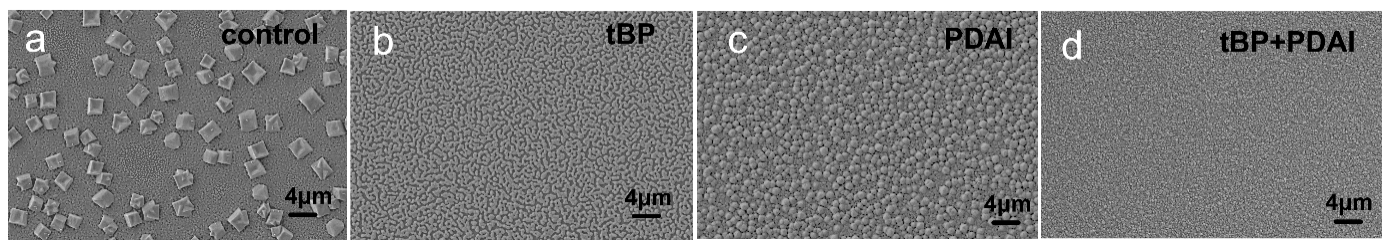


**Figure S1. SEM images of tin perovskite films** (a) control, (b) tBP, (c) PDAI and (d) tBP+PDAI on top of glass/ITO/PEDOT: PSS. The Magnitude is 4μm.


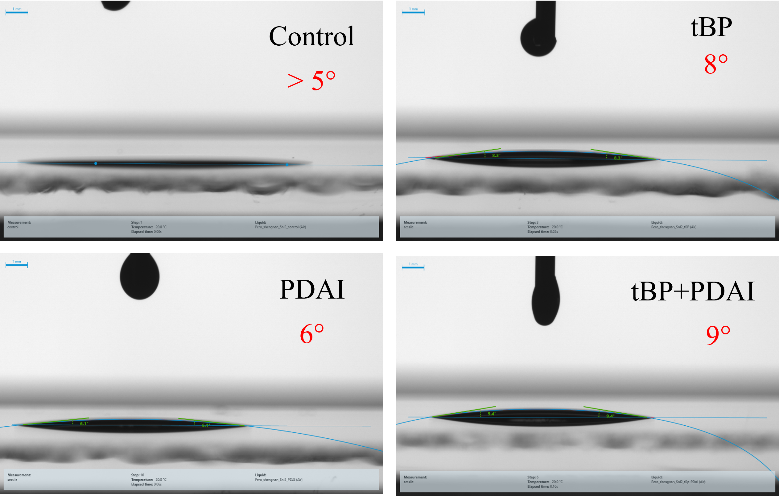


Figure S2. Contact angle measurement for perovskite inks “Control”, “tBP”, “PDAI” and “tBP+PDAI” dripped on PEDOT: PSS/ ITO substrates.


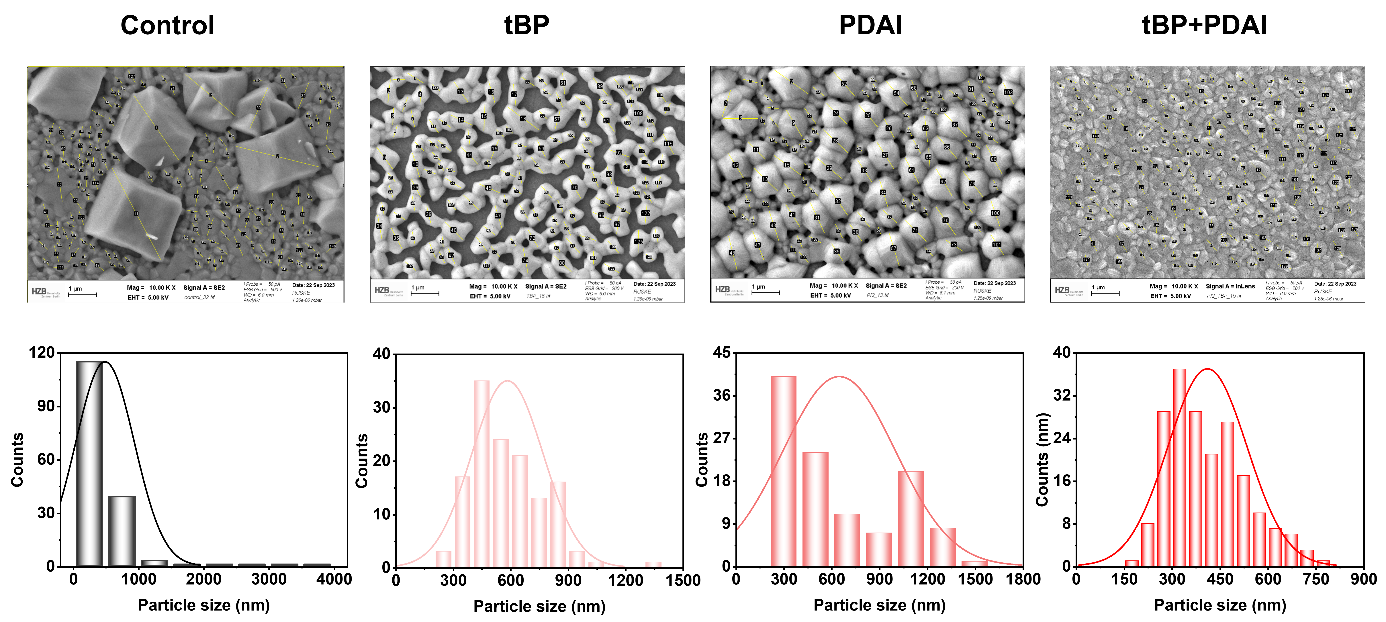


Figure S3. Grain sizes distribution mapping and statistics of grain sizes distribution for Samples “Control”, “tBP”, “PDAI”, “tBP+PDAI”. The magnitude is 10.00x.


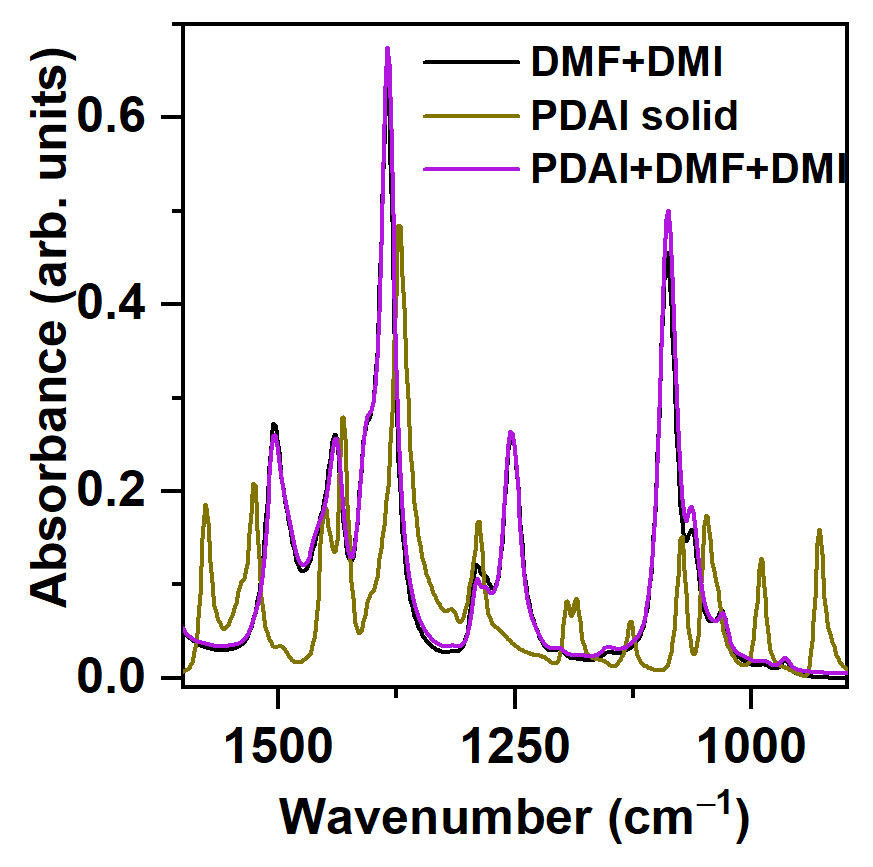


Figure S4. FTIR measurement of solvent mixture, PDAI powder and dissolved PDAI solution in DMF+DMI.


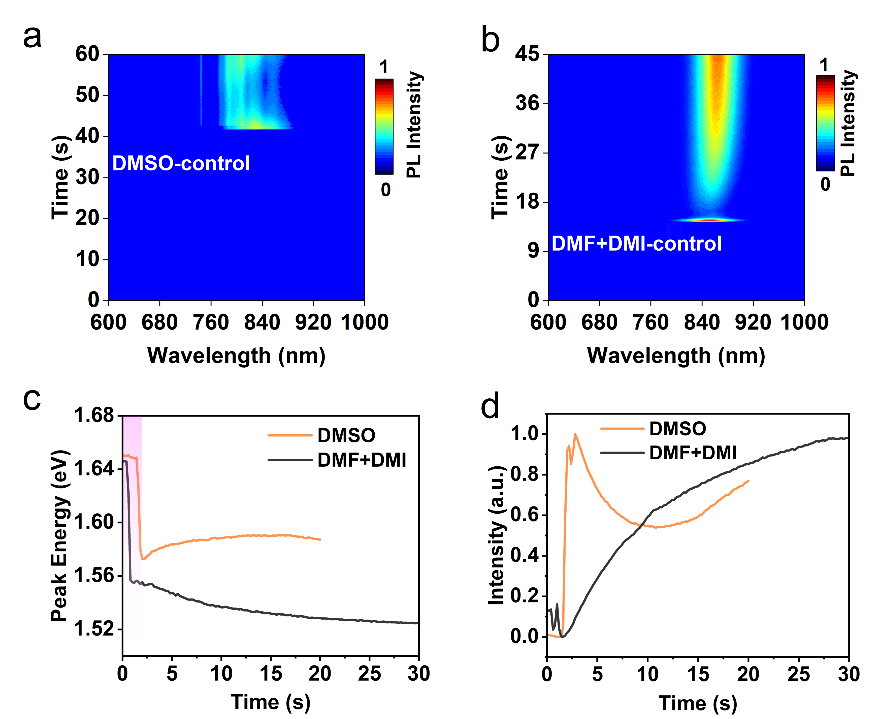


**Figure S5. Heat maps of the in-situ PL spectra** of (a) DMSO and (b) DMF+DMI processed control perovskite films; (c) the extracted values of emission peak energy and (d) normalized PL intensity from the in-situ PL measurements, x-aixs initial at anti-solvent dripping.

| Sample | control | tBP | PDAI | tBP+PDAI |
| --- | --- | --- | --- | --- |
| FWHM | 0.113 | 0.090 | 0.107 | 0.096 |
| Center | 14.096 | 14.102 | 14.077 | 14.090 |

**Table S1.** XRD (100) crystal plane fitting parameters using nonliner Gauss function.

| Sample | A_1_ | τ_1_ | A_2_ | τ_2_ | Average Lifetime (ns) |
| --- | --- | --- | --- | --- | --- |
| control | 11.8 | 17.8 | 1.2 | 53.4 | 26.4 |
| tBP+PDAI | 0.8 | 51.8 | 1.3 | 102.5 | 90.5 |

**Table S2**. TRPL fitting parameters using a bi-exponential decay function.


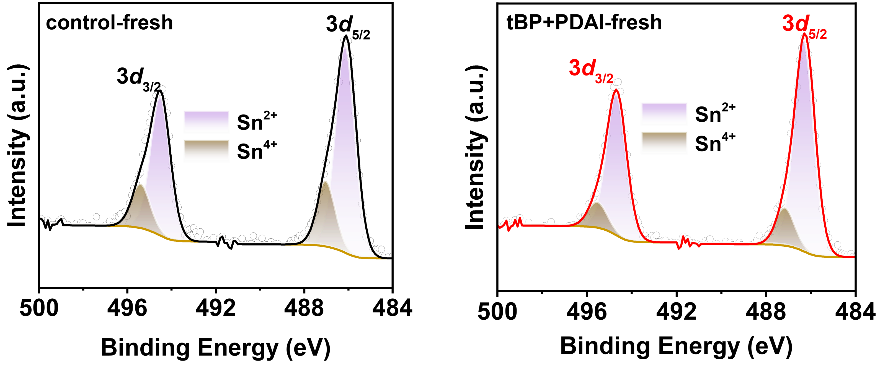


**Figure S6.** XPS spectra Sn 3*d* of fresh perovskite control and tBP+PDAI films.

|  | | Sn3*d*_5/2_ | | Sn3*d*_3/2_ | | Sn^2+/^Sn^4+^ |
| --- | --- | --- | --- | --- | --- | --- |
|  |  | Sn^2+^ | Sn^4+^ | Sn^2+^ | Sn^4+^ |  |
| control | fresh | 486.10 | 486.98 | 494.50 | 495.40 | 3.2 |
|  | exposed | 486.15 | 487.42 | 494.58 | 495.87 | 0.3 |
| tBP+PDAI | fresh | 486.26 | 487.13 | 494.70 | 495.56 | 5.7 |
|  | exposed | 486.33 | 487.21 | 494.79 | 495.64 | 1.7 |

**Table S3.** The fitting parameters of Sn3*d* peaks of Sn^2+^ and Sn^4+^.


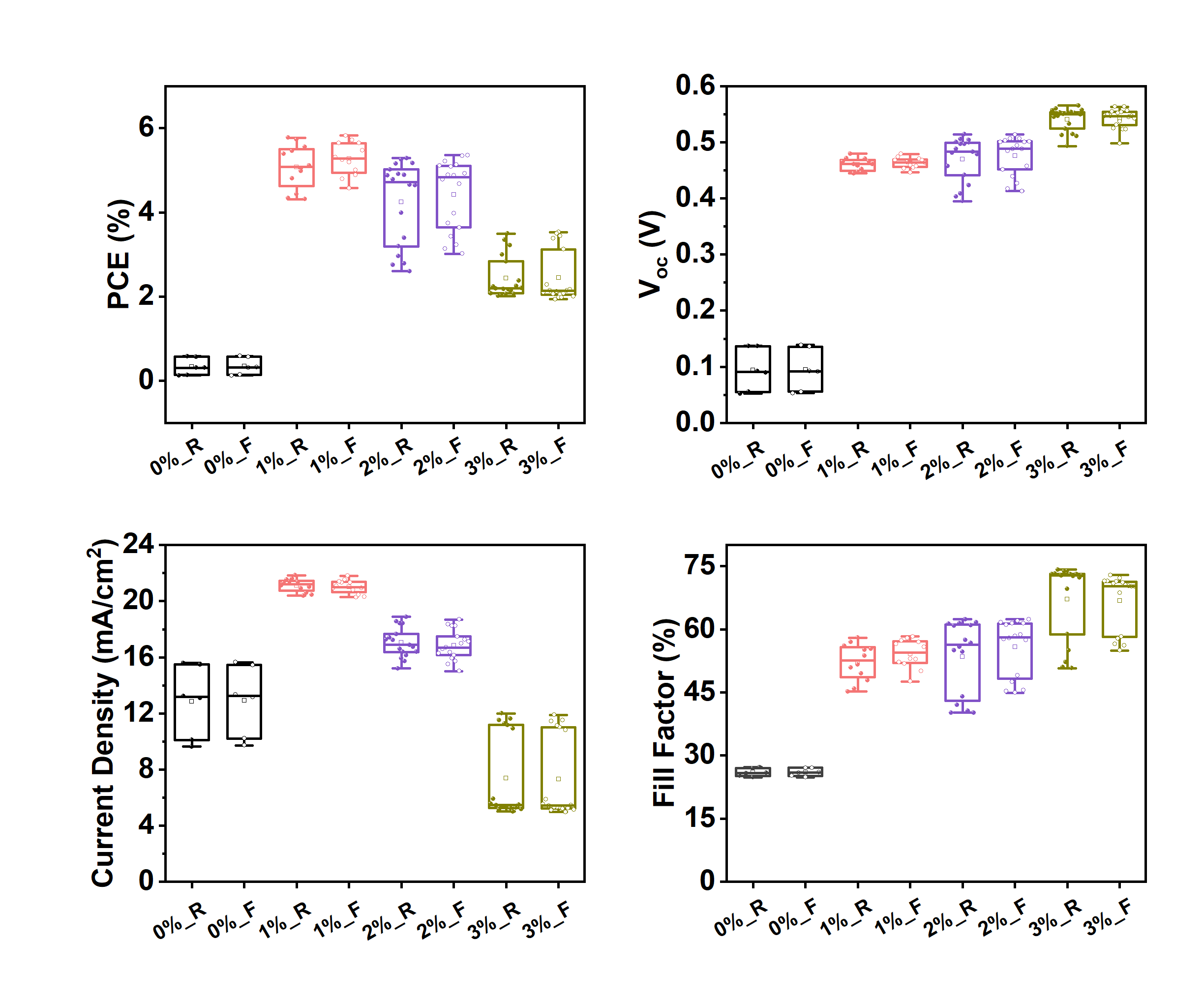


**Figure S7.** Statistical photovoltaic parameters of devices with different amount of PDAI (0%; 1%; 2%; 3%) at 50uL tBP.


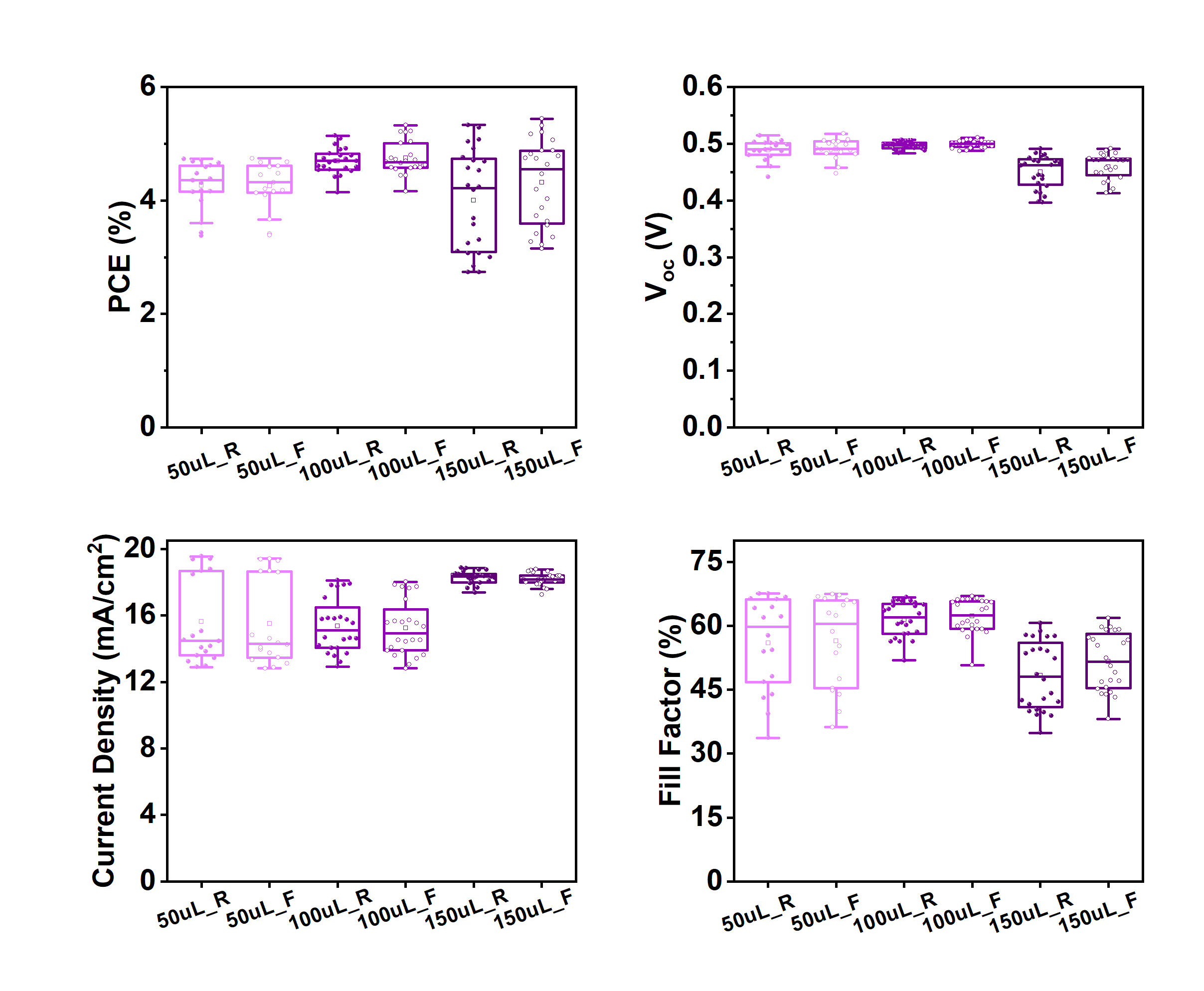


**Figure S8.** Statistical photovoltaic parameters of devices with different volume of tBP and fixed PDAI at 1%.


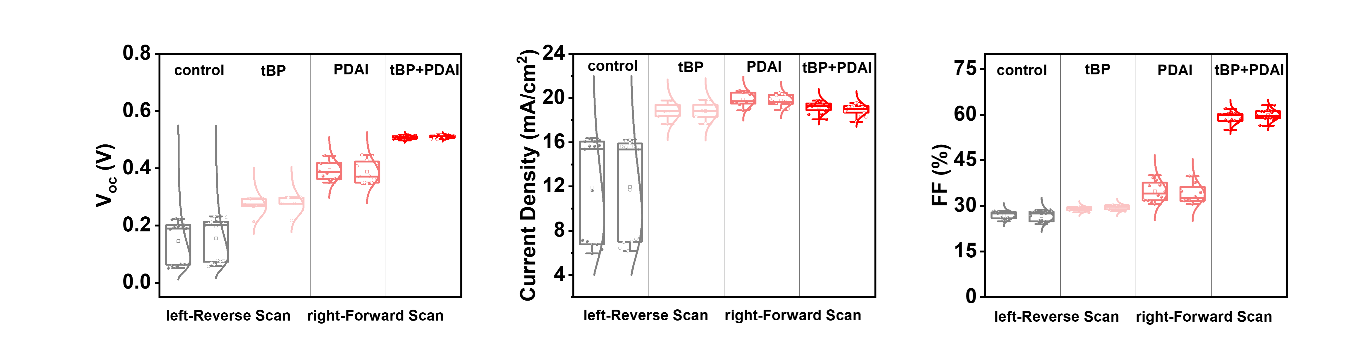


**Figure S9.** Statistical V_oc_, J_sc_ and FF parameters of devices control, tBP, PDAI, tBP+PDAI (optimised).
